# Quantifying Changes in the Cellular Thiol-Disulfide Status during Differentiation of B Cells into Antibody-Secreting Plasma Cells

**DOI:** 10.1155/2013/898563

**Published:** 2013-09-24

**Authors:** Rosa E. Hansen, Mieko Otsu, Ineke Braakman, Jakob R. Winther

**Affiliations:** ^1^Section for Biomolecular Sciences, Department of Biology, University of Copenhagen, Ole Maaloes Vej 5, 2200 Copenhagen, Denmark; ^2^Novo Nordisk A/S, 2760 Maaloev, Denmark; ^3^Cellular Protein Chemistry, Bijvoet Center for Biomolecular Research, Faculty of Science, Utrecht University, The Netherlands

## Abstract

Plasma cells produce and secrete massive amounts of disulfide-containing antibodies. To accommodate this load on the secretory machinery, the differentiation of resting B cells into antibody-secreting plasma cells is accompanied by a preferential expansion of the secretory compartments of the cells and by an up-regulation of enzymes involved in redox regulation and protein folding. We have quantified the absolute levels of protein thiols, protein disulfides, and glutathionylated proteins in whole cells. The results show that while the global thiol-disulfide state is affected to some extent by the differentiation, steady-state levels of glutathionylated protein thiols are less than 0.3% of the total protein cysteines, even in fully differentiated cells, and the overall protein redox state is not affected until late in differentiation, when large-scale IgM production is ongoing. A general expansion of the ER does not affect global protein redox status until an extensive production of cargo proteins has started.

## 1. Introduction

The cellular thiol-disulfide redox environment is defined by protein thiols (PSH) and disulfides (PS_ox_) as well as low molecular weight thiols and disulfides. In mammalian cells, by far the most abundant low molecular weight sulfhydryl molecule is glutathione (GSH). Together with its disulfide (GSSG), this pair is often referred to as the cellular thiol-disulfide redox buffer. 

In the cytosol of eukaryotic cells, glutathione is highly reducing with a ratio of GSH to GSSG of at least 3,000 [1, 2], and consequently the majority of protein cysteines are found as PSH. The high concentrations of PSH and GSH in this compartment are important in the cellular defense against thiol oxidants [3], during thiol-disulfide stress, formation of mixed disulfides between protein and glutathione (PSSG) serves as a mechanism for protecting PSH and GSH from irreversible oxidation. In contrast to cytosolic proteins, secretory proteins often contain disulfide bonds, and the glutathione redox pool in the secretory compartments of the cell is found to be considerably more oxidizing than the cytosolic pool [4]. Disulfide bond formation is an essential step for the correct folding of many secretory proteins [5], and in eukaryotic cells their folding and assembly takes place in the endoplasmic reticulum (ER). In this compartment, molecular chaperones and enzymes for disulfide bond formation and glycosylation support protein folding. The maintenance of a proper ER redox environment is crucial for the folding of secretory proteins. If the redox environment becomes too reducing, the formation of disulfide bonds is hampered [5]. If too oxidizing, folding intermediates with nonnative disulfide bonds can accumulate [6]. A number of oxidoreductases, which may have different functions and/or substrate or tissue specificities in the assistance of folding secretory proteins, are found in the ER of mammalian cells [7]. The best characterized oxidoreductase is protein disulfide isomerase (PDI), which introduces, reduces, and reorganizes disulfide bonds in a broad variety of substrate proteins [8]. The oxidative pathway remains unresolved, but PDI may be reoxidized by a number of enzymes including PDI peroxidases, GPx7 and GPx8 [9], peroxiredoxin 4, and the flavoprotein Ero1 (endoplasmic reticulum oxidoreductin 1), for review see [10, 11].

Professional secretory cells are specialized in producing secretory proteins and are characterized by their abundant ER. One example is the terminally differentiated B cell, also referred to as plasma cell, which secretes enormous amounts of antibodies, that is, immunoglobulins (Ig). While resting B cells do not secrete antibody, they do express a membrane-bound Ig on their cell surface as a subunit of the B cell receptor, which upon binding of antigen activates a signaling cascade that can lead to differentiation into antibody-secreting plasma cells. The differentiation is accompanied by many morphological changes to accommodate production of large amounts of secreted antibody. This includes a general increase in cell volume with a preferential expansion of the ER [12]. In addition, the differentiation is accompanied by dramatic changes in the proteome of the cell [13, 14]; as expected, the ER proteins are significantly up-regulated.

IgM is the first antibody produced in the adaptive immune response. IgM is typically secreted as disulfide-linked pentamers or hexamers of a subassembly consisting of two identical heavy chains (*μ*) and two light chains (*λ*). The pentameric holoprotein in addition contains a J-chain, which the hexamer does not. As each subassembly contains 16 disulfide bonds and the J-chain contributes 4 disulfide bonds, almost 100 disulfide bonds need to be formed for each secreted IgM [15]. This oxidative folding may generate reactive oxygen species (ROS). ROS production is increased during B cell differentiation and counterbalanced by a strong antioxidant response [16].

We set out to investigate how this enormous load on the secretory machinery affects the global thiol-disulfide environment of the B cell. We have applied a previously developed method for quantitative determination of the absolute levels of PSH, PS_ox_, and PSSG on all cellular proteins (including membrane proteins) in cultured mammalian cells, and combined these data with quantifications of GSH and GSSG in the same cells. In this way, we have obtained a picture of the global changes in cellular thiol-disulfide redox status during differentiation of the resting B cell into an antibody-secreting plasma cell.

## 2. Results

### 2.1. Strategy for Global Quantification of the Thiol-Disulfide Environment

**Figure 1:**
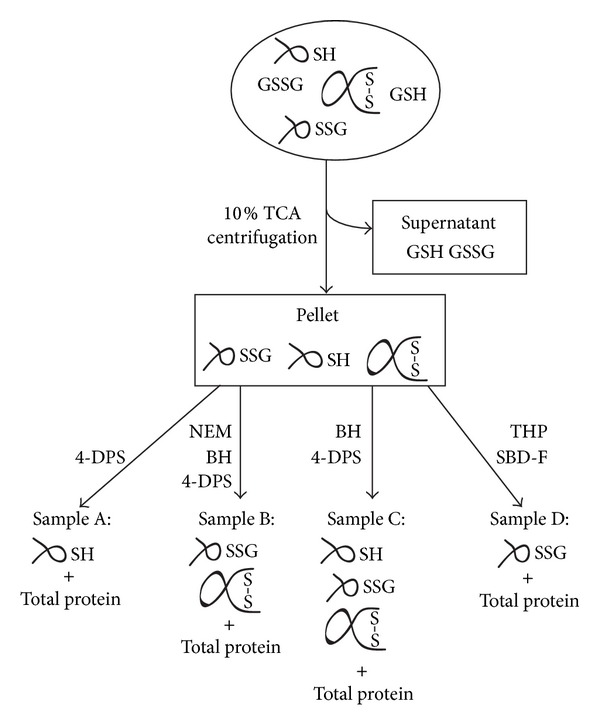
Flow chart of the experimental approach for global quantification of cellular redox species. To avoid contribution of protein disulfides from serum, cells were suspended and washed in phosphate buffered saline prior to addition of trichloroacetic acid (TCA) to 10%. After centrifugation, soluble GSH and GSSG were quantified from the supernatant while protein sulfhydryls were quantified from the pellet. The TCA pellet was divided into four samples (A-D). Sample A was directly incubated with 4,4′-dithiodipyridine (4-DPS) to quantify PSH. To quantify PS_ox_, free thiols in sample B were first alkylated with N-ethylmaleimide (NEM). Disulfides then were reduced using sodium borohydride (BH) followed by thiol quantification with 4-DPS. The main advantage of this strategy is that thiol alkylation, disulfide reduction, and thiol quantification can be performed in the same test tube as excess NEM is inactivated by BH, whereas excess BH is easily removed by the addition of acid. As a control, Total PS was measured experimentally in sample C by directly reducing disulfides with BH followed by thiol quantification with 4-DPS. Finally, PSSG in sample D was quantified by reduction of disulfides with tris(hydroxypropyl)phosphine (THP) and fluorescent labeling of thiols with 7-fluorobenzo-2-oxa-1,3-diazole-4-sulfonate (SBD-F). Selective quantification of GS-SBD derivative was performed using HPLC as described [3]. In addition to quantification of redox species, the total protein content in each pellet was quantified and used as a common denominator to compare the individual samples.

Quantitative studies of the cellular redox status involve a variety of technical challenges due to the reactive nature of the SH group. Great care must be taken to avoid artificial air oxidation and to eliminate cross-reactivity between the thiol and disulfide specific reagents, which can otherwise lead to deceptive conclusions [17]. By applying a previously developed technology that carefully considers these technical pitfalls [3], we can quantitatively determine the cellular levels of total sulfhydryl equivalents in low molecular mass thiols and in protein. The key features of the experimental approach are illustrated in Figure 1. To avoid perturbation of the cellular thiol-disulfide status during cell lysis and sample preparation, cells were acidified by the addition of TCA to a final concentration of 10%, resulting in immediate protein denaturation and precipitation. This combination of rapid trapping and deprotonation simultaneously unfolds redox enzymes, some of which have low thiol pK_a_ and are fairly acid-stable, and quenches generic thiols by protonation. To fully exploit the strength of our approach, we did not FACS-sort cells before analysis, nor did we homogenize and fractionate cells.

The TCA pellets were solubilized by sonicating in appropriate buffers with high concentrations of SDS or urea to quantify the different sulfhydryl species in all cellular proteins including membrane proteins. PSH and PS_ox_ levels were determined with a highly sensitive HPLC assay based on the thiol quantification agent 4-DPS [18]. The total value of protein cysteines (Total PS) was calculated by the addition of PSH and PS_ox_ and to verify the method, this value was also determined experimentally. For experiments performed on resting B cells, there is an excellent agreement between the experimentally determined value and the calculated sum of the experimentally determined PSH and PS_ox_ (data not shown). Finally, PSSG levels were selectively quantified by the use of the thiol derivatization agent SBD-F. The SBD-GS derivative is highly fluorescent and can be quantified specifically due to its unique retention time in an HPLC chromatogram [3]. In addition to protein sulfhydryls, the total protein content of each sample was determined and used as a common denominator to compare the individual samples. This is a crucial step as it eliminates any bias from a possible uneven division of the TCA pellet into fractions. Furthermore, the requirement for a common denominator in this study is particularly important, as it also eliminates any bias due to morphological differences between cell samples. The total protein content was quantified using a method based on complete hydrolysis in HCl followed by quantification of released amino acids with ninhydrin [3]. This constitutes a highly reproducible and sensitive method and, with a proper standard, it yields numbers that can be calibrated to “amino acids in protein.” Furthermore, the ninhydrin assay is independent of protein solubility and hence includes both soluble and membrane proteins. Thus, all the following data will be shown as sulfhydryl per amino acid (SH/aa).

### 2.2. Quantifying the Thiol-Disulfide Environment of the Resting B Cell

**Figure 2:**
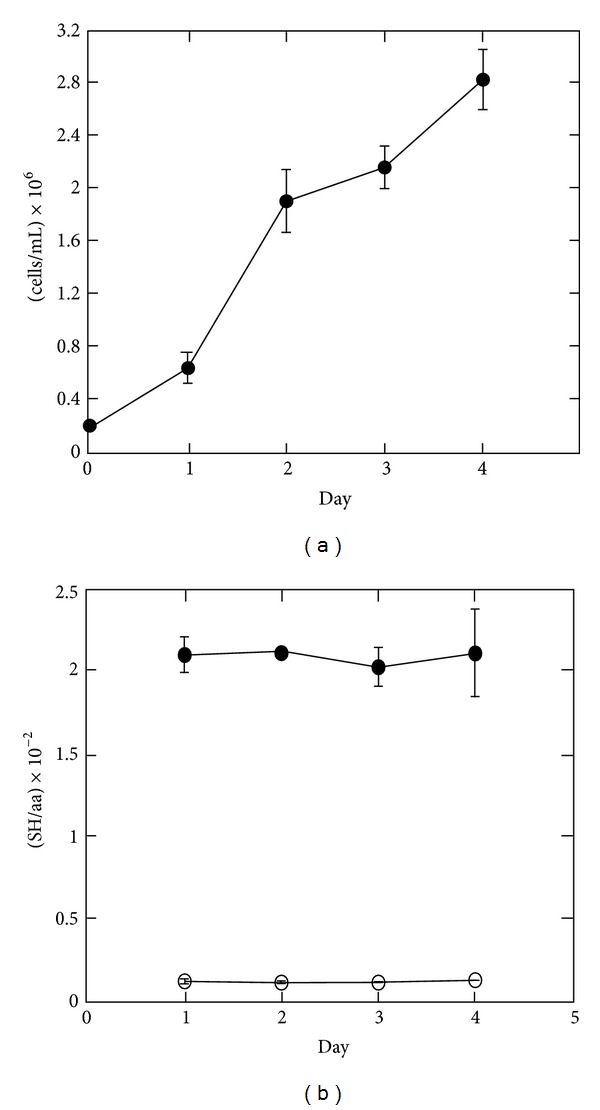
Cell density does not affect protein thiol-disulfide status in resting B cells. Experiments were performed on two independent cultures. (a) Growth curve of 1.29*μ* ^+^ lymphoma cells cultured in suspension as described in [13]. Each day a fraction of the culture was harvested for global quantification of the thiol-disulfide status, as illustrated in Figure 1. The graph shows cell densities at the times of sample preparation. (b) Distribution of protein sulfhydryl equivalents in samples harvested on days 1–4. SH/aa values for PSH (●) and PS_ox_ (○) are shown.

As a model for B cell differentiation, we used a previously established system based on the murine B cell lymphoma 1.29*μ* ^+^ which can be induced by lipopolysaccharide (LPS) to secrete IgM [13, 19]. To obtain a well-defined reference point for the differentiation of B cells into plasma cells, we quantified the thiol-disulfide status in uninduced B cells to obtain a reference point for differentiation into plasma cells. Cells were seeded to a density of 0.2 × 10^6^ cells/mL, and samples for redox quantification were taken each day during the next four days. Although cell density increased considerably during this period, the protein redox state remained constant throughout the experiment (Figures 2(a) and 2(b)). In addition, levels of PSSG, GSSG, and GSH remained constant (data not shown), and we concluded that the global thiol-disulfide status is independent of cell density. Accordingly, the mean value of data obtained for each of the redox species during the four days was calculated, and the relative distributions of the different protein and glutathione sulfhydryl equivalents are given in Table 1. From these data, we concluded that the vast majority of cellular sulfhydryl equivalents exists in the reduced thiol form with only 5% and 9% of the PS and GS equivalents engaged in disulfide bond formation, respectively. An extremely small fraction of cellular sulfhydryl equivalents is found as PSSG. Together, these data describe the total thiol-disulfide environment of resting B cells, and we used them as reference point for studying the differentiation into antibody-secreting plasma cells. In the remaining part of this study, these data will be referred to as day 0 in the differentiation. Interestingly, the distribution of thiol and disulfide equivalents in resting B cells is very similar to that of HEK (human embryonic kidney) cells, where 6% of the PS equivalents were found as PS_ox_ and 8.5% of the GS equivalents were found as GSSG. In addition, our observation that cellular PSSG levels are extremely low is supported by results in HEK and HeLa cells [3]. It should be mentioned that although cells are grown in the presence of *β*-mercaptoethanol, which was detected in the TCA supernatant and also in very small amounts in the TCA pellet, it did not interfere with the glutathione or PSSG measurements because fluorescent *β*-mercaptoethanol derivatives were separated efficiently from GS derivatives by HPLC (data not shown).

### 2.3. Changes in the Cellular Thiol-Disulfide Status Induced by Differentiation

**Figure 3:**
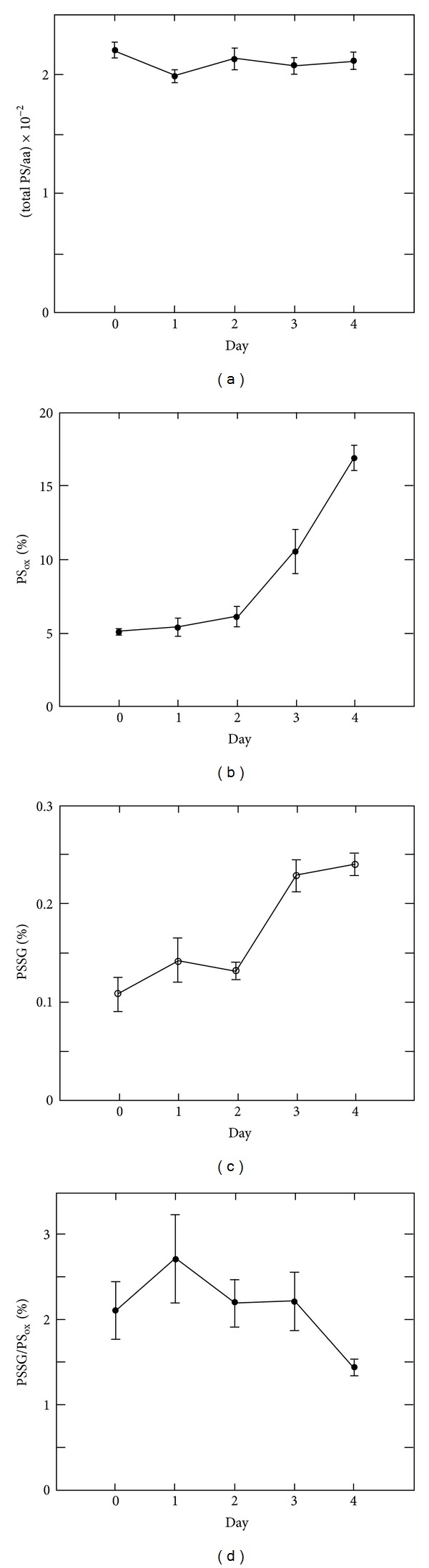
Quantitative changes in protein thiol-disulfide redox state during B cell differentiation. Protein thiol-disulfide status was measured each day during B cell differentiation in three independent cultures induced with LPS, as illustrated in Figure 1. Values are given as mean ± SEM. The values for resting B cells are represented as “Day 0.” (a) The total fraction of protein cysteines per amino acid (total PS cal). (b) Percent of PS_ox_ relative to total PS cal. (c) Percent of PSSG relative to total PS cal. (d) PSSG as a percent of PS_ox_.

**Figure 4:**
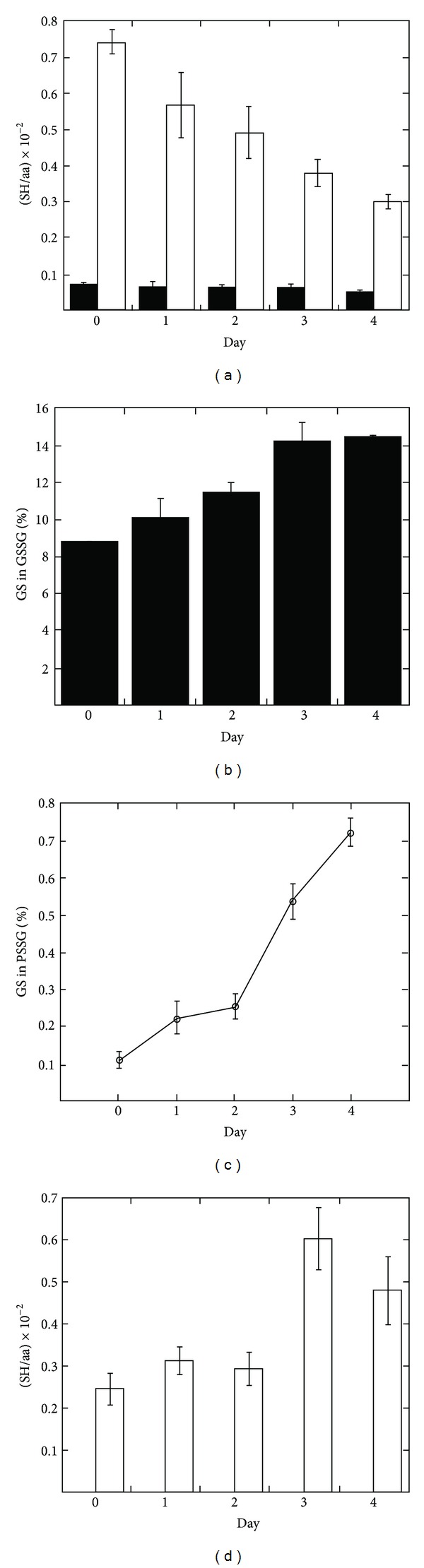
Quantitative changes in glutathione thiol-disulfide redox state during B cell differentiation. Values from experiments performed on three independent cultures are given as mean ± SEM. The values for resting B cells are represented as “Day 0.” All data are normalized to the amino acid content of the TCA precipitated cells and data processing is performed as described in the legend of Table 1. (a) SH/aa values of intracellular GSH (white bars) and (GS in GSSG) (black bars). (b) Percent (GS in GSSG) relative to (Total GS). (c) Percent (GS in PSSG) relative to (Total GS). (d) SH/aa values of GS equivalents quantified from the media.

Resting 1.29*μ* ^+^ cells were treated with LPS to induce differentiation into antibody-secreting plasma cells. Samples were prepared for global thiol quantification after 1, 2, 3, and 4 days of LPS treatment. From the SH/aa of total PS equivalents (Figure 3(a)), we find that the frequency of cysteine residues in proteins is fairly constant (~2%) throughout the differentiation. This is in excellent agreement with the experimentally determined values for other mammalian cell lines as well as calculated values for eukaryotes in general Hansen [3, 20]. The SH/aa for total PS was expected to be largely unaffected during differentiation as the IgM monomer has a cysteine frequency of 2.5% (IgM has 1,540 amino acids, of which 38 are cysteines [21]). Although the absolute value of total PS equivalents remained unchanged throughout the experiment, the distribution of protein thiols and disulfides was influenced by the LPS-induced cell differentiation. The percentage of protein thiols engaged in disulfide bond formation remained largely unaffected for two days after LPS induction, but at day three the PS_ox_ values had doubled, and a total increase by a factor of 3.3 was found at day four (Figure 3(b)). Likewise, after a lag time of two days, on day three PSSG had increased by a factor of 2.2, but in contrast to PS_ox_, the PSSG value did not increase further on day four (Figure 3(c)). Interestingly, the ratio of PSSG to PS_ox_ remained essentially unchanged throughout the experiment (Figure 3(d)). Quantification of soluble glutathione equivalents revealed that the absolute concentrations of GSSG remained largely unaffected (Figure 4(a)), but the fraction of oxidized GS equivalents (GS in GSSG) relative to total GS equivalents (total GS) increased from 8.8% to 14.5% (Figure 4(b)). This was caused by a gradual decrease in GSH (Figure 4(a)) resulting in an overall decrease of 56% in (total GS) at day 4 compared to day 0. The GS equivalents were not recovered as PSSG, which throughout the study remained a minute fraction of total GS equivalents (Figure 4(c)). To rule out that the decrease in intracellular GS was caused by an increase in dying cells, the level of apoptotic and necrotic cells was measured using flow cytometry and staining with the cell-impermeable dye propidium iodide (PI). Although the fraction of viable (PI-negative) cells decreased during the differentiation by a factor of 1.5 (Supplementary Material Figure 1 available online at http://dx.doi.org/10.1155/2013/898563), it could not account for our observed decrease in intracellular glutathione (see Supplementary Material text). As the decrease in intracellular GS was not explained by an increase in cell death, we measured whether GS equivalents were secreted by the cells. No glutathione was detectable in a blank medium sample (data not shown); media from uninduced cells, however, contained measurable amounts of glutathione, and the values increased significantly on day three and four after induction (Figure 4(d)). It should be noted that these numbers are only rough estimates and that the values are most likely underestimated due to extracellular GS degradation catalyzed by *γ*-glutamyl transferase. From the data shown in Figures 4(a) and 4(d), the increase in extracellular GS/aa equivalents at day 4 relative to day 0 was calculated to (0.23 ± 0.1) × 10^−2^ while the decrease in intracellular GS/aa was calculated to (0.46 ± 0.05) × 10^−2^. As the two numbers are in the same size range, it is possible that the differentiating cells secrete glutathione (details are given in Supplementary Material text).

## 3. Discussion

The transformation of resting B cells into antibody-secreting plasma cells involves an extensive expansion of the ER [12] and both ER resident proteins and proteins involved in redox balance are up-regulated linearly during differentiation [13]. How cells cope with this sudden increase in secretory activity has been the subject of numerous studies [22, 23]. This study, for the first time, provides a quantitative overview of the cellular thiol-disulfide status during differentiation of resting B cells into antibody-secreting plasma cells. We applied a previously developed method to quantify soluble GSH and GSSG as well as protein thiols and disulfides in cells [3]. Importantly, the method includes both soluble and membrane proteins, which precludes bias due to morphological changes during B cell differentiation. We find that the differentiation process affects the global protein thiol-disulfide status with an increase factor of 3.3 in the fraction of oxidized protein thiols at day 4 of differentiation, compared to day 0. The effects on the glutathione redox status are less significant with an increase factor of 1.6 in the fraction of oxidized GS equivalents. The changes in glutathione redox state were caused by a general depletion of GS equivalents from differentiating B cells. 

The differentiation of B cells into plasma cells has been studied in great detail at the proteomic level [13, 14]. Full IgM production is not initiated until two days after activation. Until then, the cell prepares by ensuring that metabolic capacity and secretory machinery can cope with the mass production of antibody molecules. We did not find any change in protein thiol-disulfide status until the third day of activation, when the fraction of PS_ox_ increased by a factor of 2.2. The kinetics for the change in protein redox state were identical to the kinetics for IgM production. Our results suggest that a general expansion of the ER does not affect protein redox status until an extensive production of cargo proteins is initiated. The increase of IgM production at day three is possibly preempted by a process known as “proactive” unfolded protein response (proactive UPR) [24]. The UPR is a stress signaling process that is initiated when unfolded polypeptides accumulate in the ER [25]. This process leads to an up-regulation of ER chaperones and folding enzymes which prevents ER stress. While unfolded protein stress (or ER stress) is not involved in the initial expansion of the ER in professional secretory cells [13, 14], it is essential for B cell differentiation [26, 27]. UPR-induced oxidases such as Ero1*β* may facilitate the change in protein redox state on the third day of differentiation to initiate disulfide-dependent IgM polymerization and its subsequent secretion. The role of glutathione in the ER has been a subject of intense debate. Initially GSSG was thought to provide oxidizing equivalents for disulfide bond formation, but after identification of the Ero1 proteins this hypothesis was discarded. Instead, GSH now is considered to be involved in the isomerization of nonnative disulfide bonds [28–30] to consume excess oxidizing equivalents produced by the Ero1 proteins [31] and to activate Ero1 by reducing its regulatory disulfides [32]. Consequently, the abundance of GSSG in the ER is altogether assumed to be at least partially caused by Ero1 activity. The mechanisms by which the ER maintains its GSH/GSSG redox balance are unknown. Excess GSSG could be reduced by an ER resident glutathione reductase, it could be transported to the cytosol for reduction or it could be secreted from the cells [28]. During B cell differentiation Ero1*α* is up-regulated by factors of 3.0 and 2.4 at day 3 and 4, respectively [14], and consequently we expected GSSG levels to increase. Surprisingly, GSSG levels remained constant throughout differentiation, but the overall cellular glutathione redox status did become more oxidizing gradually (Figure 4(a)). This was the result of a depletion of total GS equivalents at the expense of GSH (Figure 4(b)). There are three possible explanations for the decrease in total intracellular GS, (1) differentiation of B cells leads to secretion of GS equivalents, (2) GSH is irreversibly oxidized to sulfinic or sulfonic acids, which is not detected by the quantification method, and (3) GS equivalents are released by the fraction of PI positive cells. We found an increase in extracellular GS on days 3 and 4 of the differentiation that was of about the same magnitude as the intracellular decrease (Figure 4(d)). This result supports model (1) and suggests that GSH converted to GSSG by the up-regulation of Ero1 proteins and is exported to the media. This export could serve as a mechanism for relieving cells from any oxidative load caused by up-regulation of Ero1 proteins. We can, however, not make any certain conclusions regarding the cause of intracellular GS depletion, as the levels of extracellular GS could in principle be explained by the fraction of PI-positive cells releasing their intracellular GS content to the media (see Supplementary Material text).

Due to the extremely reducing glutathione redox potential of the cytosol, the vast majority of PSSG is expected to be found in the oxidizing compartments of the cell [3]. In a study of liver microsomes, 50% of the GS equivalents were found as PSSG [33], suggesting that a major fraction of the glutathione in the ER is associated with protein. However, this fraction was subsequently estimated to be significantly lower (i.e., less than 2% PSSG) based on whole-cell quantification with the assumption that no PSSG is found in the cytosol [3]. Under the same assumption; that is, that all PSSG equivalents are found in the ER and that the concentrations of GS equivalents are the same in all cellular compartments, we can estimate that the maximal fraction of PSSG is relative to total GS equivalents in the ER, in fully differentiated B cells. The ER volume is reported to constitute at least 10% of total cell volume in antibody-secreting B cells [12] and, accordingly, maximally 7% (0.7%/10%) of the GS equivalents in ER are found as PSSG. Thus, even in highly active secretory cells, PSSG only constitutes a minor fraction of total GS equivalents of the ER. These results support the notion that the ER glutathione redox environment is more reducing than previously assumed [34]. It is generally assumed that the level of GSSG is critical for the amounts of PSSG formed [35]. Interestingly, we found that the ratio of PSSG to PS_ox_ was independent of differentiation (Figure 3(d)), suggesting that the oxidizing compartments of the cell maintain a constant level of PSSG, and consequently, that the ER glutathione redox status is tightly regulated throughout differentiation. This may be explained by the activation of oxidative stress during the early stage of B cell differentiation and followed by a strong antioxidant response [16, 36]. Maintenance of a proper ER glutathione redox environment can be a crucial factor in securing the correct folding of IgM. In this study, we have for the first time characterized the changes in thiol-disulfide state during differentiation of B cells. In general, the differentiation does not cause massive thiol-disulfide stress to the cells. The steady state levels of PSSG are maintained at very low levels, even in fully differentiated cells, and the overall protein redox state is not affected until late in differentiation, when large-scale IgM production has started and the ER stress response has been activated.

## 4. Materials and Methods

### 4.1. Cell Culture and Activation of B Cells

1.29*μ* ^+^ cells were maintained in suspension as described in [13] and after 2 days, cell culture medium was replaced by fresh medium. For differentiation, cells were cultured in the presence of 20 *μ*g/mL LPS (Sigma). Three independent cultures were induced with LPS and samples were taken after 1, 2, 3, or 4 days of differentiation. As a control, samples from 3 independent cultures grown without LPS were collected as well. Each day after LPS activation, samples were collected for flow cytometric analysis. Cells were stained with PI (Sigma) and flow cytometry data were obtained with FACScalibur (BD Biosciences) and analyzed using Cellquest software (BD Biosciences).

### 4.2. Quantifying Intracellular Redox Species

Cells were harvested by centrifugation followed by a wash step with 10 mL Dulbecco's 1x PBS (PAA laboratories) to eliminate traces of protein from the media. Cells were resuspended in 1 mL ice-cold 10% (w/v) TCA and incubated on ice for 30 minutes followed by centrifugation. The supernatant, used for quantification of GSH and GSSG, was immediately frozen in N_2_ and kept at −80°C until later use. The TCA pellet, used for protein thiol quantification, was washed in 10% TCA by four cycles of sonication and centrifugation. Before the final centrifugation step, the suspension was divided in four to quantify PSH, PS_ox_, PSSG, and total PS. The PSH, PSSG, and total PS samples were immediately frozen in N_2_ and kept at −80°C until later use. The PS_ox_ sample was directly solubilized and alkylated by sonicating the pellet in 500 *μ*L of 5% SDS, 1 mM EDTA, 20 mM NEM (Sigma) in 0.5 M Tris-Cl pH 8.3. The alkylation was allowed to proceed for at least 20 min before the sample was transferred to −80°C. Thiol and disulfide species in TCA supernatant and TCA pellet fractions were quantified as described in [3]. Briefly, PSH samples were solubilized in 5% SDS, 1 mM EDTA in 0.4 M sodium citrate, pH 4.5 and protein thiols were quantified by the addition of 4-DPS (Sigma) to a final concentration of 0.5 mM followed by HPLC analysis. For quantification of PS_ox_, samples alkylated with NEM were reduced by the addition of BH (Sigma) to a final concentration of 3.3% (w/v). Prior to quantification with 4-DPS, BH was destroyed by the addition of HCl, as described in [18]. Total PS was quantified by solubilizing the pellet in 5% SDS, 1 mM EDTA, and 0.5 M Tris-Cl pH 8.3, reducing all thiols with BH, followed by thiol quantification with 4-DPS. The PSSG sample was solubilized in 6 M urea, 8 mM EDTA, and 200 mM bicine pH 9.2. Disulfides were reduced with 2.1 mM THP (Calbiochem) and thiols were derivatized by the addition of 6.4 mM SBD-F (Fluka) for 1 hour at 60°C. To compare samples, total protein content was determined by using a ninhydrin based assay [3].

### 4.3. Quantifying Intracellular and Secreted Glutathione

An HPLC assay based on thiol derivatization with N-(1-pyrenyl)maleimides (Fluka) was used to quantify GSSG and total GS (GSH + GSSG) from the supernatant as described in [1]. GSH was quantified by subtracting oxidized glutathione from total glutathione. For quantification of secreted glutathione, proteins in media from harvested cells were precipitated by the addition of TCA to 10% (v/w) and incubated on ice for 30 minutes followed by centrifugation. Total glutathione was quantified from the TCA supernatant as described above.

## Supplementary Material

Supplementary material includes a short evaluation of the possible influence of dying cells on the intracellular and extracellular levels of glutathione.

## Figures and Tables

**Table 1:** Relative distribution of protein and glutathione sulfhydryl equivalents in resting B cells.

	Protein^a^	Glutathione^b^
% Thiols	95 ± 4	91 ± 5
% Disulfides^c^	5.1 ± 0.2	8.8 ± 0.5
% PSSG^d^	0.11 ± 0.02	0.15 ± 0.02

Values are given as the means ± SDM.

^a^Percentage of measured SH/aa relative to total protein SH/aa.

^b^Percentage of measured SH/aa relative to total glutathione SH/aa. Total glutathione equivalents are calculated by addition of the SH/aa values of [Total soluble GS], quantified from the TCA supernatant, and [GS in PSSG], quantified from the TCA pellet, according to Figure 1. GSH is calculated by subtracting [GS in GSSG] from [Total soluble GS].

^c^Values are calculated as SH/aa equivalents engaged in disulfide bond formation.

^d^Values are calculated as SH/aa equivalents engaged in PSSG formation.

## Abbreviations

GSH: Glutathione
GSSG: Glutathione disulfide
PSH: Protein thiols
PS_ox_: Protein disulfides
PSSG: Mixed disulfides between protein and glutathione
ER: Endoplasmic reticulum
PDI: Protein disulfide isomerase
Ero1: Endoplasmic reticulum oxidoreductin 1
Ig: Immunoglobulin
TCA: Trichloroacetic acid
SBD-F: 7-fluorobenzo-2-oxa-1,3-diazole-4-sulfonate
THP: Tris(hydroxypropyl)phosphine
NEM: N-ethylmaleimide
BH: Sodium borohydride
4-DPS: 4,4′-dithiodipyridine
LPS: Lipopolysaccharide
ROS: Reactive oxygen species.
