# Supplementary material for: Quantifying Changes in the Cellular Thiol-Disulfide Status during Differentiation of B Cells into Antibody-Secreting Plasma Cells
Source: Int J Cell Biol. 2013 Sep 24;2013:898563. doi: 10.1155/2013/898563 (PMC3800581; doi:10.1155/2013/898563)
Supplement: Supplementary file 1 — Supplementary material includes a short evaluation of the possible influence of dying cells on the intracellular and extracellular levels of glutathione. [file 898563.f1.pdf]

Supporting information

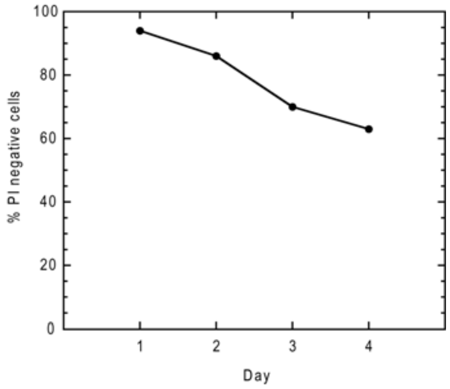

SI Fig. 1: Ratio of PI-negative cells during B cell differentiation. Each day during LPS activation samples were collected for flow cytometric analysis. Apoptotic and necrotic cells were stained with PI and flow cytometry data were obtained with FACScalibur and analyzed using Cellquest software.

Is intracellular GS decrease caused by dying cells?

To determine whether the measured decrease in intracellular GS was caused by the decrease in PI negative cells, we estimated what the expected decrease would be if the fraction of dying cells had lost all their GS content.

The total amount of soluble GS equivalents at day 0 were determined to (0.8 ± 0.03) x 10^{-2} SH/aa.

At day four, 63% of cells were PI negative and the expected number of GS is:

0.8 x 10^{-2} SH/aa x 0.63 = 0.5 x 10^{-2} SH/aa.

As the experimentally determined number of GS at day four is (0.35 ± 0.02) x 10^{-2}, we can conclude that the decrease in intracellular GS is not accounted for by loss of GS from dying cells.

Is extracellular GS increase caused by PI positive cells?

To determine whether the measured increase in extracellular GS was caused by the increase in dying cells, we estimated what the expected increase would be, if the fraction of PI positive cells had lost all their GS content.

As described above, the total amount of soluble GS equivalents at day 0 ((0.8 ± 0.03) x 10^{-2} SH/aa) is used for the calculation.

At day four, 37% of cells were PI positive, and the expected number of GS released to the media is:

0.8×10^{-2} SH/aa x 0.37 = 0.3×10^{-2} SH/aa.

The experimentally determined increase in media GS content at day four relative to day 0 is (0.23 ± 0.1) x 10^{-2} SH/aa. Thus, we cannot exclude that the increase in extracellular GS content is caused by leakage from PI positive cells.
